# Patterns of genetic divergence in the Rio Grande cooter (*Pseudemys gorzugi*), a riverine turtle inhabiting an arid and anthropogenically modified system

**DOI:** 10.1093/jhered/esae011

**Published:** 2024-02-19

**Authors:** Michael W Vandewege, Javier Gutierrez, Drew R Davis, Michael R J Forstner, Ivana Mali

**Affiliations:** Department of Clinical Sciences, College of Veterinary Medicine, North Carolina State University, Raleigh, NC, USA; Biomedical Forensic Sciences, Anatomy and Neurobiology Department, Boston University Chobanian and Avedisian School of Medicine, Boston, MA, USA; Department of Biology, Eastern New Mexico University, Portales, NM, USA; Biodiversity Collections, Department of Integrative Biology, The University of Texas at Austin, Austin, TX, USA; Department of Biology, Texas State University, San Marcos, TX, USA; Fisheries, Wildlife, and Conservation Biology Program, Department of Forestry and Environmental Resources, North Carolina State University, Raleigh, NC, USA

**Keywords:** arid landscapes, Emydidae, migration, population genomics, RADSeq, Rio Grande

## Abstract

The lower Rio Grande and Pecos River of the southwest United States have been heavily modified by human activities, profoundly impacting the integrity of their aquatic wildlife. In this context, we focused our study on the population genomics of the Rio Grande Cooter (*Pseudemys gorzugi*), a freshwater turtle of increasing conservation concern, residing in these two rivers and their tributaries. The genetic data revealed two distinct populations: one in the Pecos and Black Rivers of New Mexico and another in the Rio Grande and Devils River of Texas, with admixed individuals identified at the confluence of the Rio Grande and Pecos River. In addition to having a smaller geographic range, we found lower observed heterozygosity, reduced nucleotide diversity, and a smaller effective population size (*N*_e_) in New Mexico population. Our results depict a significant isolation-by-distance pattern across their distribution, with migration being notably infrequent at river confluences. These findings are pivotal for future conservation and restoration strategies, emphasizing the need to recognize the unique needs of each population.

## Introduction

The lower Rio Grande and its primary tributary, the lower Pecos River, are vital watercourses flowing through the rugged Chihuahuan Desert, North America’s second-largest desert. This region is renowned for the richness and distinctiveness of its flora and fauna (Briggs et al. 2020), however, the river systems have been heavily modified by human activities, rendering them among the most endangered in the American Southwest. The construction of numerous dams and channels, beginning in the late 19th century, has drastically changed the natural dynamics of both rivers (Dearen 2016). Coupled with increased water extraction for industries like oil and gas, this has led to reduced flood frequency, diminishing water quantity and quality, exacerbating the impacts of climate change (Jensen et al. 2006; Collins and Rosen 2020; Walker et al. 2021; Scanlon et al. 2022; Williams et al. 2022). Among the many man-made alterations affecting these rivers, the Red Bluff Reservoir is a particularly notable example, having significantly altered the downstream habitat of the Pecos River and made it inhospitable for several aquatic organisms (Hoagstorm 2009; Pease and Delaune 2021; Mahan et al. 2022). Studies have revealed an enduring pattern of impacts on population genetic health, including aspects like genetic divergence and diversity loss, directly attributable to the environmental stressors adversely affecting the river’s ecosystem (Galindo et al. 2016; Osborne et al. 2021).

Given these environmental changes, even the most enduring taxa like turtles, renowned for their longevity, ability to persist in polluted environments, and high starvation tolerance, can face significant challenges (Lovich et al. 2018; Buchanan et al. 2019; Mahan et al. 2022). Turtles are an evolutionary success given their survival through mass extinctions, diversity, and distribution, but currently find themselves among the most imperiled vertebrate groups in the Anthropocene (TTWG 2021). The combination of their longevity, late maturity, and resiliency can mask recruitment gaps, complicating the identification of population declines (Browne and Hecnar 2007; Howell et al. 2019). Additionally, their extended generation times and slow mutation rates often result in delayed genetic consequences related to habitat alteration. In the context of the Chihuahuan Desert’s aquatic fauna, the Rio Grande Cooter (*Pseudemys gorzugi*) stands out as a prime example of a chelonian grappling with the repercussions of habitat degradation and fragmentation.

**Fig. 1.**
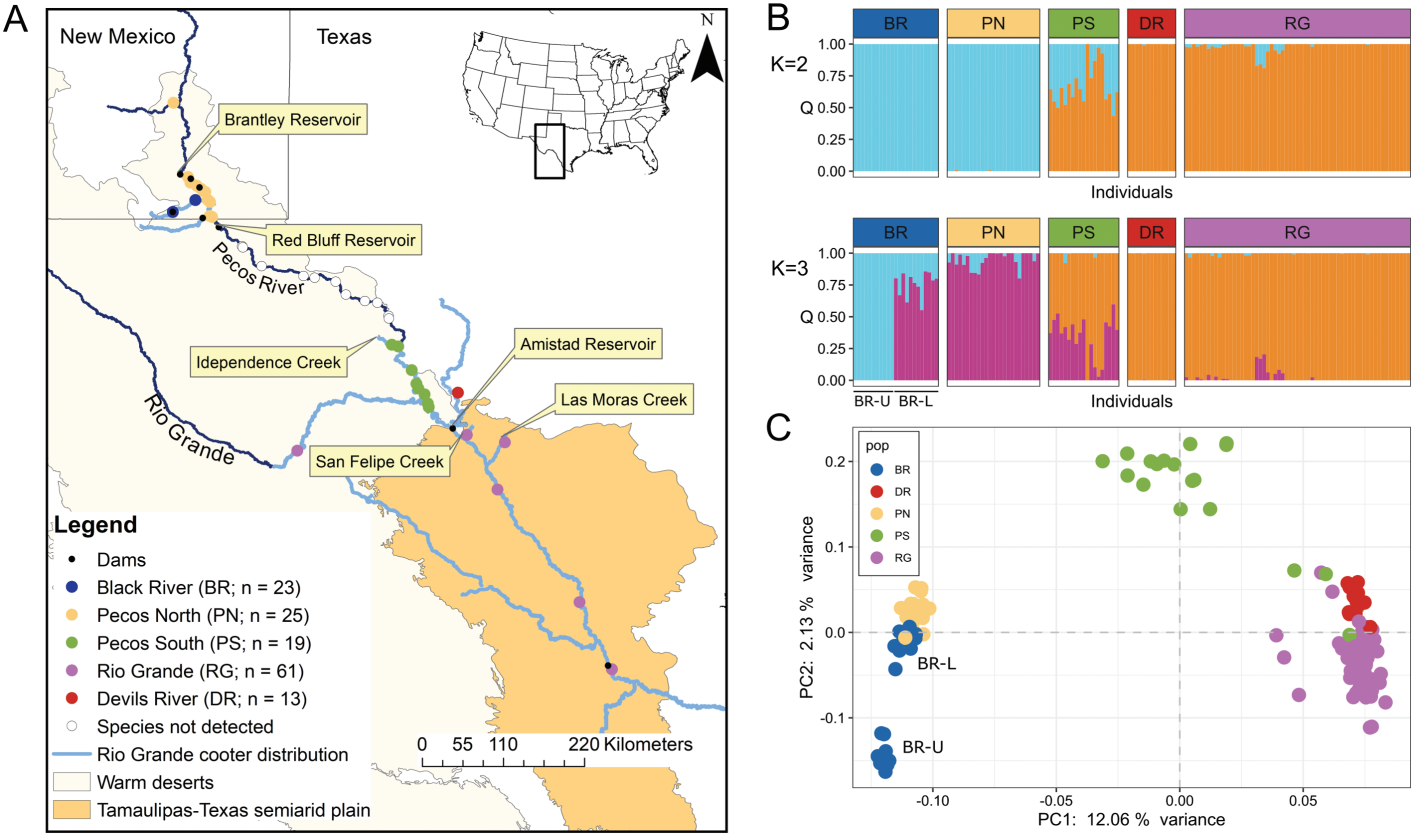
A) Map of the range of *P. gorzugi* in the United States and Mexico. Dark blue lines denote the major rivers and light blue lines denote the distribution of *P. gorzugi*. We color coded sampling localities for the Black River (BR), Pecos North (PN), Pecos South (PS), Devils River (DR) and Rio Grande (RG). Empty circles denote where sampling efforts failed to detect the species. B) NGSadmix plots for K = 2 and K = 3. Each bar on the X axis represents an individual within an a priori group and the Y is the probability an individual belongs to a New Mexico (light blue) or Texas (orange) population. BR-U and BL-L indicate samples that came from the Black River upstream and Black River downstream, respectively. C) A PCA generated from covariances calculated by PCAngsd.

Among the freshwater turtle species that inhabit both the Rio Grande and Pecos River, *P. gorzugi* has the most restricted range, almost entirely confined within the Chihuahuan Desert and Tamaulipas-Texas Semiarid Plain ecoregions (Fig. 1A). Although generally considered a riverine turtle, many have alluded to the affinity of *P. gorzugi* for smaller tributaries, clear spring runs, and deep, slow-moving pools in the streams it occupies (Degenhardt et al. 1996; Ernst and Lovich 2009; Dixon 2013; Legler and Vogt 2013). Globally listed as Near Threatened by the International Union for Conservation of Nature (van Dijk 2011), *P. gorzugi* receives limited protection in New Mexico and Mexico, where it is listed as threatened (SEMARANT 2010; NMDGF 2020), and it is considered a species of conservation concern in Texas (TPWD 2012). The species was reviewed for potential federal listing under the US Endangered Species Act (ESA; USFWS 2021), but the proposal was ultimately rejected (USFWS 2022). Regardless of its conservation status, the sustainability of *P. gorzugi* is inextricably linked to the health of the aquatic ecosystems in these arid landscapes, with its survival being directly threatened by the same environmental factors that imperil the rivers it inhabits.

A population genetic survey using the mitochondrial ND4 gene and microsatellite markers found limited genetic differentiation across the range of *P. gorzugi* in the United States suggesting high connectivity (Bailey et al. 2008). However, this has not been a consistent result among other chelonians that occur across the Chihuahuan Desert. For example, the Rio Grande endemic *Trachemys gaigeae* has experienced a contemporary loss in connectivity and reduced gene flow between populations in New Mexico and Texas (Jackson 2010; Forstner et al. 2014). Similarly, freshwater turtles (i.e. *Terrapene coahuila*, *Trachemys taylori*, and *Apalone atra*) in Coahuila, México, have experienced isolation and loss of genetic diversity resulting from drying conditions and the subsequent loss of habitat connectivity (McGaugh 2012; Cortés-Rodríguez et al. 2021).

*P. gorzugi* has long been considered one of the least-studied freshwater turtle species in the United States (Lovich and Ennen 2013). However, due to the proposal for federal listing under the ESA, *P. gorzugi* has garnered increased research attention (e.g. Mali et al. 2018; Bogolin et al. 2021; Suriyamongkol et al. 2022). Mahan et al. (2022) surveyed for *P. gorzugi* throughout the lower Pecos River, failing to detect the species over a ~390 km stretch of river downstream from the Red Bluff Reservoir (Fig. 1A), and attributed these findings to the river’s heightened conductivity levels. Recent range-wide survey initiatives in the United States have yielded additional samples beyond the range examined by Bailey et al. (2008) and genomic analyses have drastically improved. Restriction-site associated DNA sequencing (RADSeq) yielding single nucleotide polymorphisms (SNPs) have become the most economic and accessible genetic markers that offer enhanced resolution of genetic diversity and population structure compared to microsatellites (Georges et al. 2018; Xie et al. 2019; Gallego-García et al. 2021). Here, we sought to improve our understanding of *P. gorzugi* range-wide genetic diversity and population structure amid a rapidly changing environment and identify the most vulnerable populations.

## Methods

### Sample collection

Sample (tissue or blood) collection occurred opportunistically during various *P. gorzugi* ecology projects (Fig. 1A; *n* = 141). Surveys for turtles were conducted either via snorkeling or hoop-net traps. Recent intensive survey efforts occurred in the Black River (BR; *n* = 23), a tributary of the Pecos River in Eddy County, New Mexico, from 2016 to 2022 (Mali et al. 2018; Letter et al. 2019; Suriyamongkol and Mali 2019). These efforts were expanded to the lower Pecos River in 2020 and 2021 (Mahan et al. 2022) which increased the geographical area coverage. However, it is important to reiterate that extensive survey efforts did not detect the species along a ~390 km stretch downstream from the Red Bluff Reservoir (Mahan et al. 2022). For that reason, we refer to the Pecos River samples north of Red Bluff Reservoir as Pecos North (PN; *n* = 25) and samples south of Independence Creek as Pecos South (PS; *n* = 19). Five *P. gorzugi* collected from Chaves County, New Mexico (~80 km upstream from Brantley Dam), represent the northernmost locality of the species range (Suriyamongkol et al. 2020), were included in the PN group. In addition to the Black and Pecos rivers, we included samples from a single location on the spring-fed Devils River (DR; *n* = 13), the Rio Grande itself and two smaller tributaries where the species can be locally abundant (i.e. San Felipe Creek and Las Moras Creek) (RG; *n* = 61) (Bogolin et al. 2021; Bassett et al. 2022). Most samples were collected between 2016 and 2021. Two exceptions are samples collected from Big Bend National Park in 2005 and samples from San Felipe Creek collected in 1995 and 2010.

### DNA preparation, sequencing, and mapping

DNA was extracted from blood or tissue samples using a QIAGEN DNeasy Blood and Tissue Kit guided by the manufacturer’s protocol. NGS QC and sequencing were performed by Admera Health. Specifically, a Qubit 2.0 was used to ensure more than 200 ng of DNA was present prior to library prep. DNA was digested with MluCI and SphI restriction enzymes and size selected for 350 bp after adapter ligation (Parham et al. 2020). Illumina libraries were prepped following guidelines in Peterson et al. (2012) and sequenced on an Illumina NovaSeq 6000 S4 platform for 150 bp paired-end reads. Paired-end reads were cleaned with process_radtags 2.6 provided by Stacks v2 (Rochette et al. 2019). Specifically, we removed any reads containing ambiguous base calls (N), low-quality base calls (average Phred score < 10, as specified in process_radtags) and reads that lacked SphI or MluCI cut sites. Paired-end reads were mapped to a reference genome from a closely related taxon, the Painted Turtle (*Chrysemys picta*) (GCA_000241765.2), using BWA mem (Li and Durbin 2010) with default parameters. Overall, the average per sample coverage was approximately 3X at RAD loci and at such low sequencing depths, confidence in individual genotypes is low, particularly at heterozygous sites. To address this, we used ANGSD 0.94 (Korneliussen et al. 2014) to account for genotype uncertainty and calculated genotype likelihoods for variable sites on chromosomal scaffolds longer than 100 kb.

### Population structure

For analyses related to population structuring we used the SAMTOOLS genotype likelihood model (-GL 1) in ANGSD, required a minimum nucleotide Q score of 20 (-minQ 20), a minimum MapQ score of 30 (-minMapQ 30); we retained variants with a minimum minor allele frequency (MAF) of 0.01, set a SNP_pvalue equal to 1e-6, retained sites that were present in 60% of samples with individual sequencing depths between 2 and 25X (-setMinDepthInd 2 and -setMaxDepthInd 25) and output genotype likelihoods in BEAGLE format (-doGLF 2). Since many downstream analyses make assumptions about the independence of genomic loci, we used ngsLD (Fox et al. 2019) to calculate linkage and pruned sites using the supplied prune_ngsLD.py script with a min weight of 0.2 and max distance of 25 kb since linkage disequilibrium (LD) dropped below 0.1 well within 25 kb (Supplementary Figure 1). To quantify population structure, we used NGSadmix (Skotte et al. 2013) to estimate the admixture proportions of individuals. We ran NGSadmix for each K between 1 and 5 for 10 iterations each and selected the best K by finding the largest change in log likelihood between Ks (ΔK) (Evanno et al. 2005). We also generated a covariance matrix from unlinked genotype likelihoods using PCAngsd 1.2 (Meisner and Albrechtsen 2018) and performed a PCA on covariances using the *eigen* function in R 4.3.2.

### Genetic diversity measurements

Samples were a priori divided into five groups based on the origin of the samples in the Rio Grande Basin system: Pecos River North (PN), Pecos River South (PS), Black River (BR), Devils River (DR), and Rio Grande (RG; Fig. 1A). To calculate genome-wide heterozygosity, we created a folded site frequency spectrum (SFS) for each individual using ANGSD. Specifically, we created a saf file (-doSaf 1) using reads with a -minMapQ 30, and sites with -minQ 20. We removed tri-allelic variants (-skipTriallelic 1), kept reads that mapped to one location (-uniqueOnly 1), computed base alignment qualities (-baq 2), and adjusted mapping qualities for excessive mismatches (-C 50). We retained uniquely mapping reads (-uniqueonly 1) and read pairs with correctly mapped mates (-only_proper_pairs 1) and included the remove_bads filter. We generated a folded SFS from the saf file with realSFS that is included with ANGSD and calculated observed heterozygosity (*H*_O_) by dividing the number of heterozygous sites by the total. We also generated population level folded SFSs using the above parameters while retaining sites present in more than 60% of the samples for each population. We calculated nucleotide diversity (*Pi*) for each group using thetaStat (Korneliussen et al. 2013) based on Watterson’s theta (*θ*_w_) over 10 kb windows. We also used realSFS to generate 2D folded SFS between all pairs of populations to calculate pairwise *F*_ST_.

### Migration inference

From the genotype likelihood dataset, we measured pairwise genetic distances among individuals using NGSdist 1.0 (Viera et al. 2015) as raw p-distances and calculated pairwise river distances using the R package *riverdist* (Tyers, 2023). We estimated a correlation between genetic and geographic distances using a Mantel test in the R package *ade4* with 9,999 permutations. Further, we used EEMS (Petkova et al. 2016) to visualize migration surfaces along river habitats to identify corridors and barriers to gene flow. EEMS identifies deviations from exact IBD using a stepping-stone model (Kimura and Weiss 1964) between adjacent demes. We used the pairwise genetic distances calculated by NGSdist as input and used Google Maps API v3 (www.birdtheme.org/useful/v3tool.html) to create a habitat file that bordered our samples along the Pecos River and Rio Grande. We adjusted parameters to ensure the recommended 10%–40% acceptance rates to help with MCMC convergence. We ran EEMS using a deme size of 500 for three independent iterations. Each run consisted of 10,000,000 iterations, sampling every 20,000 iterations after an initial 4,000,000 burn-in. We used the rEEMSplot R package (Petkova et al. 2016) to plot migration surfaces and considered migration statistically significant if the posterior probability (Pr) of effective migration rates (m) was greater than 90%.

### Demographic inferences

We estimated a contemporary effective population size (*N*_e_) for the Pecos River and Rio Grande populations excluding the admixed individuals in PS using NeEstimatorV2 (Do et al. 2014). We used ANGSD -doGeno 1 to call genotypes at the variable sites described prior to LD pruning in the Population structure section of the Methods. We filtered for sites that were present in 90% of samples and used the LD method to estimate *N*_e_ and 95% confidence intervals for all MAF thresholds (0.05, 0.02, and 0.01). We also took advantage of the folded SFS generated above and used Stairway Plot v2 (Liu and Fu 2015, 2020) to infer historical changes in *N*_e_ in both populations. We used a mutation rate of 1.28e-8 substitutions per site per generation (Shaffer et al. 2013; Green et al. 2014; Fitak and Johnsen 2018), a 20 year generation rate (Ernst and Lovich 2009) and skipped the singletons bin since errors in low coverage data could inflate the number of singletons.

## Results

### Population structuring

One hundred and fifty samples were selected for this study to cover the majority of the species range in the US. We successfully sequenced 141 of these samples (Fig. 1A; Supplementary Table 1). Between 590,262 and 1,496,779 paired-end reads survived the initial process_radtag filtering per sample, and over 99% of those reads successfully mapped to the *Chrysemys picta* reference nuclear genome. We retained 37,781 variable sites after filtering for quality and linkage pruning via ngsLD. The K with the largest deltaK was K = 2 (Supplementary Figure 2A) which largely organized samples by major rivers (Rio Grande and Pecos River). Given the substantial disjunction along the Pecos River and the predominant clustering of members into “state-based” rivers, we will refer to these as the Texas (including DR, RG, and PS) and New Mexico (including BR and PN) (Fig. 1A). Population assignment was mixed in PS section of the Pecos River, between the Independence Creek and the confluence with the Rio Grande. Samples from PS were mostly assigned to the Texas population with admixture from the New Mexico population (Fig. 1B). The ΔK method is often biased towards K = 2 even when more structuring is present (Janes et al. 2017). In fact, K = 3 model did suggest additional structuring driven by division in the Black River (Fig. 1B), but K = 4 did not have any more explanatory power (Supplementary Figure 2B). Based on the PCA analyses of our data, the first two principal components explained 14.2% of the variation (Fig. 1C). Consistently, samples from BR and PN formed a New Mexico cluster, samples from RG and DR formed a Texas cluster, and PS samples were more similar to the Texas population but exhibited intermediate distance between Texas and New Mexico clusters on the first axis (Fig. 1C). BR samples were further divided into an upstream group (BR-U) and downstream group (BR-L) on the second axis.

### Genetic diversity and differentiation

**Fig. 2.**
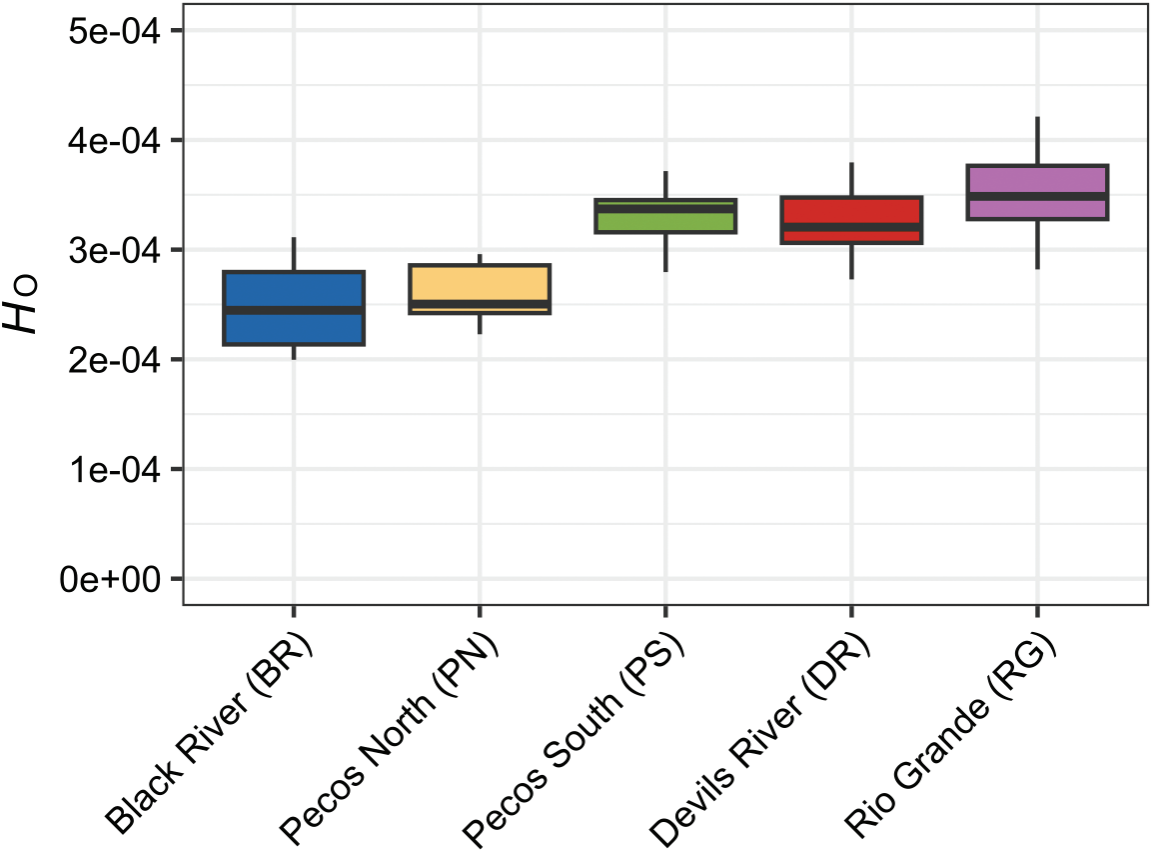
The range of observed heterozygosity (*H*_O_) among individuals in each a priori population.

We aimed to compute genetic diversity estimates for each pre-defined population to consider nuances among groups, which may be pertinent for tailoring state-specific conservation measures. Genome-wide *H*_O_ ranged between 2 and 4 × 10^−4^, which was slightly lower than *H*_O_ measured from other threatened turtles (Gallego-García et al. 2021). Heterozygosity was lowest in the New Mexico groups and the PS group was more comparable with the Texas groups (Fig. 2). We generated a distribution of *Pi* from 10 kb windows and found values were lowest in the New Mexico groups and slightly higher in the Texas groups, but nucleotide diversity in PS was similar to the BR and PN groups (Supplementary Figure 3). We generally found that pairwise *F*_ST_ exhibited low to moderate differentiation among groups and ranged between 0.00 and 0.24 (Table 1). Generally, adjacent groups (PN and BR; RG and DR; RG and PS) exhibited lower population divergence compared to geographically distant populations, and PS had higher similarity to DR and RG than PN or BR. The global *F*_ST_ between two populations defined by NGSadmix (excluding the admixed PS individuals) was 0.22 and the New Mexico group had lower *H*_O_ and *Pi* than the Texas group (Supplementary Figure 4).

**Table 1.** Global *F*_ST_ values estimated from pairwise 2D SFS for all pairs of *P. gorzugi* a priori populations.

	BR	PN	PS	RG	DR
BR	-	…	…	…	…
PN	0.031	-	…	…	…
PS	0.178	0.143	-	…	…
RG	0.239	0.211	0.053	-	…
DR	0.220	0.190	0.028	0.000	-

### Isolation by distance and dispersal patterns

**Fig. 3.**
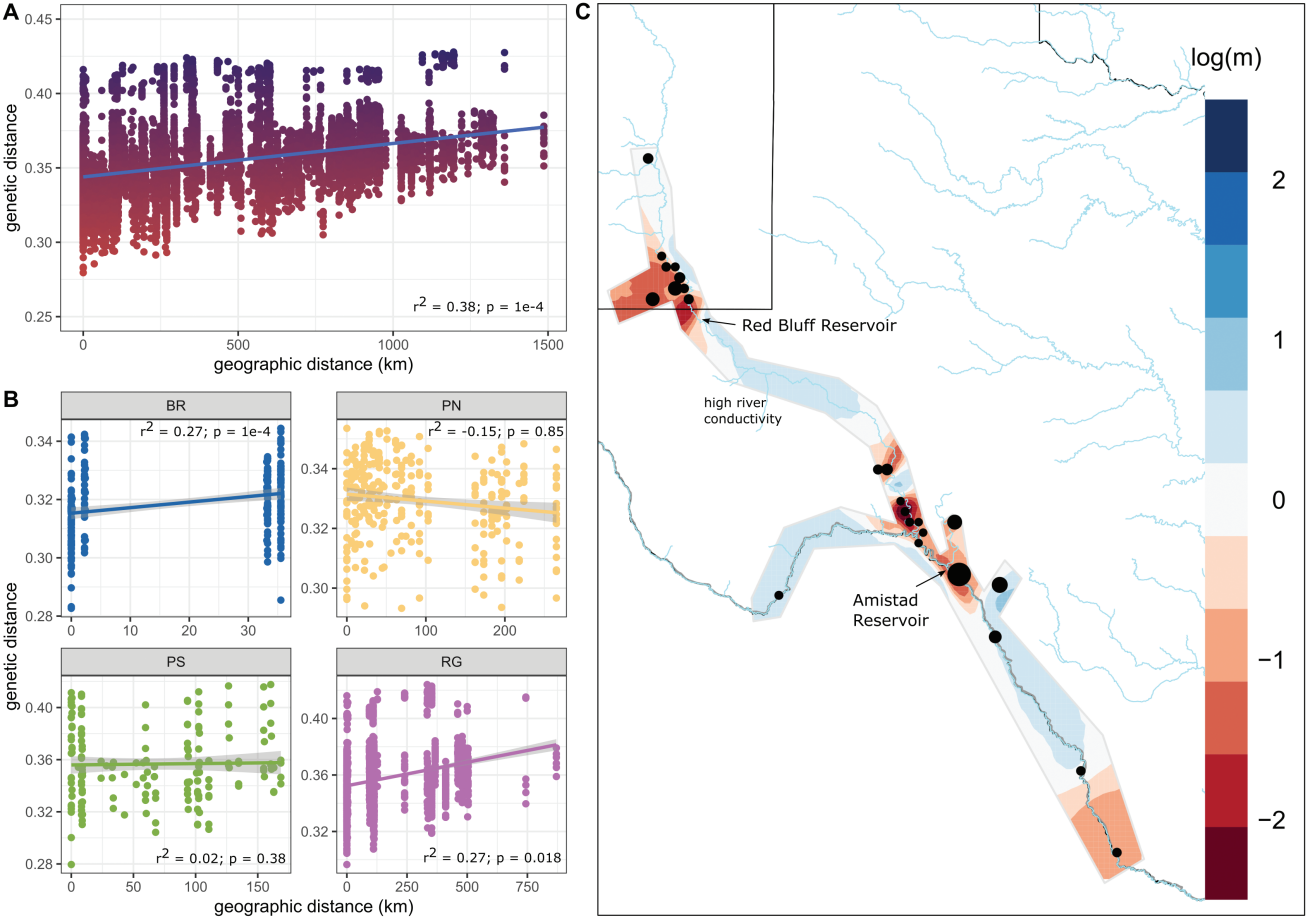
A) A scatterplot between genetic and geographic distance for all individuals, colors scale with the Y axis. Correlation and significance were measured using a Mantel randomization test. B) Correlation between genetic and geographic distance for each a priori group except DR. C) Effective migration rates estimated by EEMS throughout the sampled range. Areas where migration is higher than average are in tones of blue and where areas are lower than average are in tones of red.

Since pairwise *F*_ST_ increased with geographic distance among the a priori groups we formally measured the strength of IBD using Mantel tests. Overall, there was a strong IBD effect throughout the species range (*r*^2^ = 0.38; *p* = 0.0001) (Fig. 3A). We also measured IBD within a priori groups, excluding the Devils River (DR) because those samples were collected from one locality, and found variable strengths of IBD (Fig. 3B). We found evidence of IBD within the Black River and the Rio Grande. In contrast, the Pecos North stretch exhibited negative IBD (although not significantly different from 0), and IBD was absent in the Pecos South group.

Although EEMs did not identify any specific regions functioning as significant migration corridors, they revealed barriers to gene flow at the confluence of tributaries (Fig. 3C), however, the posterior probability of effective migration did not exceed 0.9 across most of the sampled area. Interestingly, the posterior probability only surpassed 0.9 at the junctions of rivers and tributaries, where migration rates were below average (Supplementary Figure 5). According to EEMs, IBD between PN and PS was in line with the average for the species, suggesting the genetic distance was concordant with geographic distance (Fig. 3C).

### Effective population size

**Fig. 4.**
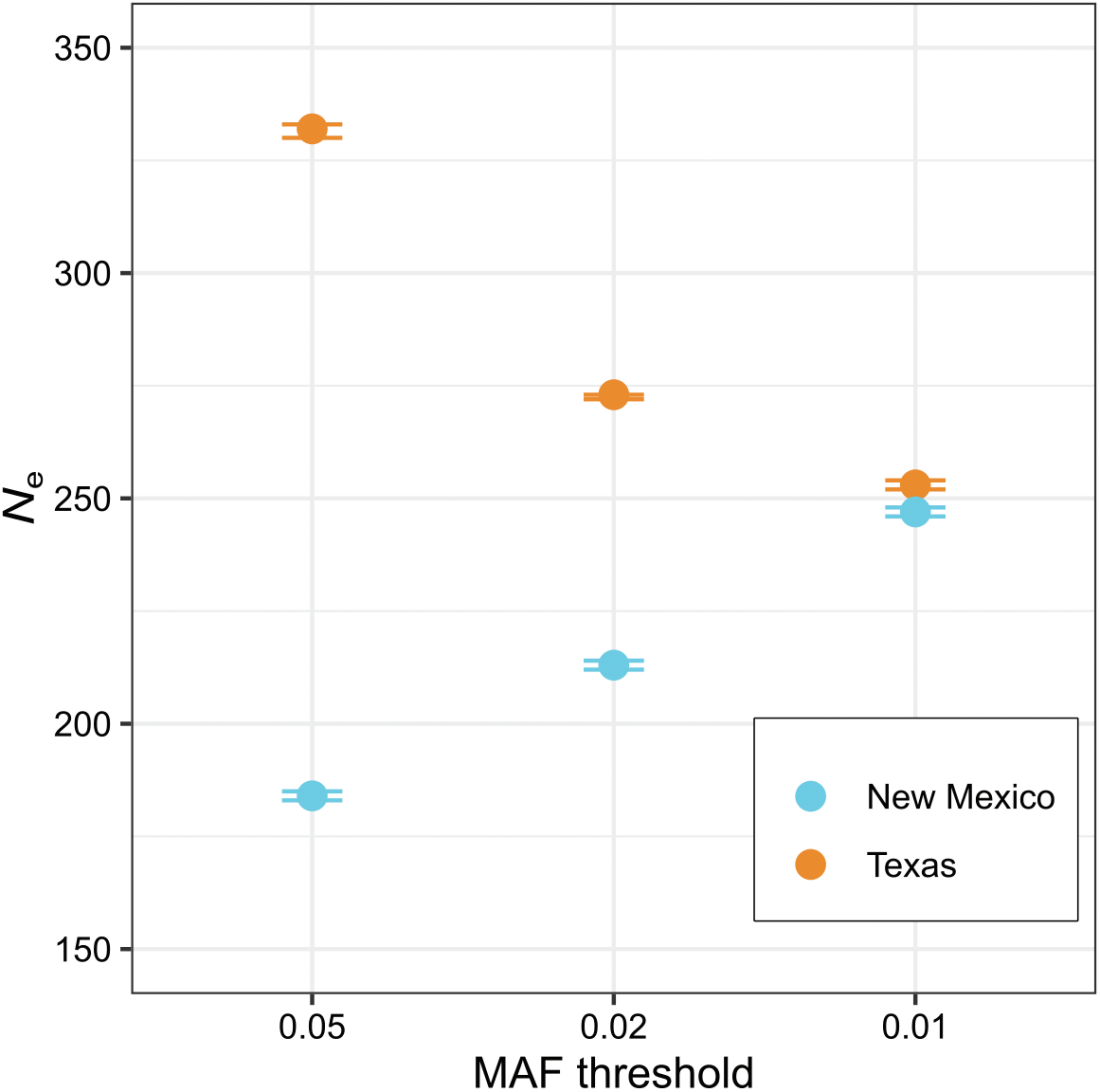
Contemporary estimates of *N*_e_ for different MAF thresholds based on LD. Error bars reflect 95% confidence intervals.

Lastly, we estimated and compared *N*_e_ between the New Mexico and Texas populations. Based on the LD method, Texas had a larger *N*_e_ than New Mexico, but *N*_e_ converged to 250 using a MAF threshold of 0.01 (Fig. 4). Analysis using the Stairway plot revealed that the effective population size (*N*_e_) was consistent in both populations in the ancient past but experienced a decline approximately 500,000 years ago. The Texas population exhibited a less pronounced drop and subsequently attained a higher *N*_e_. In contrast, the New Mexico population experienced a more substantial decline and did not recover to the same extent as its Texas counterpart (Supplementary Figure 6). We should note that *N*_e_ is a particularly difficult metric to estimate and can be influenced by sample size, sampling scheme and data type and sequencing depth (Reid and Pinsky 2022), so exact numbers should be interpreted with caution. However, both NeEstimator and Stairway plot reveal a larger *N*_e_ in Texas than New Mexico, lending additional support to this inference.

## Discussion

Our tissue sample collection provided coverage across the *P. gorzugi* range in the US (from the southernmost to the northernmost known localities), covering approximately 70% of the total species range. While the species is also found in the Rio Grande tributaries of northern Mexico (i.e. Rio San Juan and Rio Salado-Rio Sabinas) (Pierce et al. 2016), we were unable to collect or obtain samples from these areas. Nonetheless, we were able to answer critical questions related to genetic diversity, population structuring, effective population size, and isolation by distance. Our methods were more sensitive and advanced compared to those used in the previous population genetic assessment (Bailey et al. 2008), offering a deeper insight into the genetic health of the species.

Our study reveals a notable genetic differentiation between *P. gorzugi* populations above the Red Bluff Reservoir and those in the remaining species range, presenting a contrast to the findings from mtDNA haplotype analyses (Bailey et al. 2008). Yet, this pattern aligns with other aquatic taxa sharing similar geographical distributions. For instance, both the Rio Grande Shiner (*Notropis jemezanus*) and Speckled Chub (*Macrhybopsis aestivalis*) show genetic divergence between populations in the lower Pecos River in New Mexico and the Rio Grande in Texas (Osborne et al. 2021). Similarly, the Texas Hornshell Mussel (*Popenaias popeii*), which shares its habitat with *P. gorzugi* and is listed as federally endangered, demonstrates a pronounced genetic split between its New Mexico population and those in Texas (Inoue et al. 2015). Inoue et al. (2015) also observed genetic homogeneity in *P. popeii* throughout the Rio Grande and Devils River, overall, analogously to the patterns seen in *P. gorzugi*.

There were measurable genetic differences between the Texas and New Mexico *P. gorzugi* populations. Observed heterozygosity was lower, nucleotide diversity was lower, and estimates of *N*_e_ were lower in the New Mexico population. Given the smaller geographical range, physical isolation, reduced genetic diversity, and lower *N*_e_, the New Mexico populations are at greater risk to extirpation than populations in the Rio Grande. Additionally, our study uncovered fine-scale population structuring in the Black River. Parts of the Black River run underground between our surveyed upper (BR-U) and lower (BR-L) segments, which likely influences this structuring. Admixture, PCA, and isolation by distance (IBD) results indicate restricted turtle migration across this relatively short river section. This finding raises further concerns for the turtles inhabiting BR-U (Fig. 1B); their physical and genetic isolation from both the BR-L and PN turtles renders them particularly susceptible to environmental changes and underscores the importance of this area in the overall conservation priorities for this turtle.

In this study, five samples came from a location 80 km north of the generally accepted *P. gorzugi* range (Berrendo Creek, Chaves County, NM) (Fig. 1A). We expected a positive and significant IBD signal in PN given their physical distance and separation from the main population, but this was not the result. In fact, the correlation was negative although not significantly different from zero (Fig. 3B), suggesting higher similarity to their downstream counterparts than expected. We hypothesize the presence of these individuals could be attributed to anthropogenically driven dispersal (e.g. turtles escaped captivity or intentionally released) or to a natural flood facilitating an upstream dispersal event, although we found little evidence based on river gage data. There are two prior records in this region: Bundy (1951) reported *P. gorzugi* from Bitter Lake National Wildlife Refuge (BLNWR), deemed questionable by Degenhardt et al. (1996), and a verified carcass from BLNWR found in 2008 (Giermakowski and Pierce 2016). However, the Berrendo creek population, located near the BLNWR, was only recently discovered (Suriyamongkol et al. 2020). The Pecos River north of Brantley Dam to Berrendo Creek has not been surveyed for the species due to accessibility and water availability issues. Future surveys of this region would help gain a clearer picture of their natural range and the genetic inferences.

The construction of the Red Bluff Reservoir on the Pecos River in 1936, primarily for irrigation purposes, significantly altered the natural flood regime (e.g. decrease in flood frequency) and led to a marked increase in water salinity both in the reservoir and downstream (Hoagstorm et al. 2010). This change exerted extreme pressures on the aquatic life in the Pecos River (Hoagstrom et al. 2010; Pease and Delaune 2021: 2-19). While salinity tolerances for *P. gorzugi* remain largely unassessed, Seidel (1975) demonstrated that *Apalone spinifera* from the Pecos River exhibits a greater ability to concentrate urea compared to its Rio Grande counterparts, suggesting adaptation to brackish environments. Four species of *Pseudemys* have been observed in brackish waters (reviewed in Agha et al. 2018). In Florida, for example, *Pseudemys nelsoni* is known to occur in and tolerate brackish waters, however, the elevated salinity levels are seasonal, and the species has access to abundant freshwater refuges (Dunson and Seidel 1986). While the Pecos River is known for its naturally higher salinity levels due to rich gypsum deposits, diminished flows have led to prolonged unnaturally high concentrations, possibly outpacing the evolutionary adaptation capabilities of the local turtle populations (Mahan et al. 2022). The genetic differences between PN and PS populations align with what is expected given their geographic distance and PS turtles exhibit admixture with the isolated New Mexico population. These results provide evidence that the hiatus region below the Red Bluff Reservoir was once habitable or at least permitted some gene flow to the lower Pecos River region (i.e. PS). This hiatus likely represented a genetic gradient between the distinct populations in Texas and New Mexico. However, the inability to detect *P. gorzugi* in both recent and previous extensive survey efforts has hindered our understanding of the extent and boundaries of population admixture throughout the lower Pecos River in Texas.

In addition to Red Bluff Reservoir, Amistad Reservoir was constructed in 1969 to provide irrigation water storage, flood control, and hydropower generation, and collects water from the Rio Grande, Pecos, and Devils rivers. EEMs designated migration barriers that were congruent with these major reservoirs, but the reservoirs are also directly adjacent or in close proximity to tributary coalescence (Fig. 3C), so it is difficult for us to determine if these barriers are driven by natural or anthropogenic causes. However, given the long generation time and slow mutation rates of turtles, we speculate river modifications have not been present long enough to be driving this signal in the data. Nonetheless, as long as the New Mexico and Texas populations are separated, we can expect these populations to become genetically more discrete with time and this effect is directly linked to construction of the Red Bluff Reservoir. The Amistad Reservoir, while supporting numerous fish and turtle species, including *P. gorzugi*, may pose an additional barrier to movement. Impoundments in the Rio Grande have contributed to the shrinkage of the suitable habitat, population fragmentation, and extirpation of lotic species like the Texas Hornshell (Karatayev et al. 2018) and Osborne et al. (2021) suggested populations of Rio Grande Shiner and Speckled Chub upstream and downstream of Amistad Reservoir could genetically diverge in the future. Although Amistad Dam may not impede turtle movement to the same extent as it does for fish, we should be aware of potential future consequences.

While riverine turtles are known for their extensive movements, the documentation of this behavior in *Pseudemys sp.* has only emerged in recent studies (MacLaren et al. 2017; Johnston et al. 2018). The underlying reasons for why and when they make such long movements remains poorly understood, but some evidence suggests it is related to temporal variation in hydrology (Johnston et al. 2018). Our findings, supported by strong evidence of IBD (Fig. 3A), suggest that while long distance dispersal in *P. gorzugi* may be common (Sirsi 2021), gene flow itself likely occurs over short distances and can be hindered by even small stretches of land (i.e. Black River upper and lower segments), which hints at evidence of site fidelity. It is noteworthy that the majority of our Rio Grande samples (72%) were collected from two clear water creeks, San Felipe (*n* = 29) and Las Moras (*n* = 15), where *P. gorzugi* appears locally abundant. The rest of the samples (*n* = 17) were collected from four sites on the main stem of the river (Fig. 1). Although the density of *P. gorzugi* in the main stem of Rio Grande is likely considerably lower compared to clear springs and pristine rivers (i.e. Devils River), our study suggests that these turtles use the Rio Grande as a movement corridor, navigating between localized preferred habitats.

A case in point is the San Felipe Springs population. Dixon (2013) reported its near extirpation in 1998, followed by a recovery by 2010. Our combination of historic and contemporary samples allowed us to explore whether the “modern” population at San Felipe Springs originated from migrants from the Rio Grande or descended from the “historic” San Felipe turtles. Interestingly, the modern San Felipe samples exhibited slightly higher heterozygosity, although nucleotide diversity remained largely unchanged (Supplementary Figure 7). This suggests that at least some modern turtles likely migrated into the springs from the main river between 1998 and 2010. This finding highlights the critical role of habitat connectivity in this river system for the conservation of *P. gorzugi* populations.

## Conclusions

Overall, this study sheds light on the population dynamics of *P. gorzugi*, providing valuable insights to guide both the New Mexico Department of Game and Fish and the Texas Parks and Wildlife Department in their management priorities and revising conservation status assessments. This guidance is crucial, especially since *P. gorzugi* does not benefit from federal protection. In formulating future management strategies, it is vital for agencies to consider the ongoing mega-drought affecting the southwestern US and its impact on the habitats and climate change adaptation capacities of freshwater turtles. For conservation planning, it is imperative to categorize *P. gorzugi* into at least two distinct Management Units, each requiring tailored conservation strategies specific to their regions within the Rio Grande basin. We consider the habitat downstream of the Red Bluff Reservoir as unlikely to be restored to conditions suitable for *P. gorzugi*, and conservation efforts should instead concentrate on the remaining areas of their habitat. Importantly, habitat recovery options (e.g. invasive species reductions, riparian management, or restoration, alongside maintaining instream flows) provide complementary benefits to the other fauna already listed as endangered or threatened in the system.

## Supplementary Material

Supplementary material is available at http://www.jhered.oxfordjournals.org/.

esae011_suppl_Supplementary_Figures_1-7

esae011_suppl_Supplementary_Table_1

## Data Availability

Raw sequencing reads can be found under SRA Bioproject PRJNA964000. The code used in this study can be found at github.com/mike2vandy/gorzugi.

## References

[CIT0001] Agha M , Ennen JR, Bower DS, Nowakowski AJ, Sweat SC, Todd BD. Salinity tolerances and use of saline environments by freshwater turtles: implications of sea level rise. Biol Rev. 2018:93:1634–1648. doi: 10.1111/brv.12410 29575680

[CIT0002] Bailey LA , Dixon JR, Hudson R, Forstner MRJ. Minimal genetic structure in the Rio Grande cooter (*Pseudemys gorzugi*). Southwest Nat. 2008:53:406–411. doi: 10.1894/gc-179.1

[CIT0003] Bassett LG , Mali I, Nowlin WH, Foley DH, Forstner MRJ. Diet and isotopic niche of the Rio Grande cooter (*Pseudemys gorzugi*) and syntopic red-eared slider (*Trachemys scripta elegans*) in San Felipe Creek, Texas, USA. Chelonian Conserv Biol. 2022:21:199–211. doi: 10.2744/ccb-1556.1

[CIT0004] Bogolin AP , Davis DR, Kline RJ, Rahman AF. A drone-based survey for large, basking freshwater turtle species. PLoS One. 2021:16:e0257720 34705839 10.1371/journal.pone.0257720 PMC8550609

[CIT0006] Briggs MK , Lozano-Cavazos EA, Poulos HM, Ochoa-Espinoza J, Rodriguez-Pineda JA. The Chihuahuan Desert: a binational conservation response to protect a global treasure. Encycl World’s Biomes. 2020:126–138. doi: 10.1016/B978-0-12-409548-9.11966-9

[CIT0007] Browne CL , Hecnar SJ. Species loss and shifting population structure of freshwater turtles despite habitat protection. Biol Conserv. 2007:138:421–429. doi: 10.1016/j.biocon.2007.05.008

[CIT0008] Buchanan SW , Kolbe JJ, Wegener JE, Atutubo JR, Karraker NE. A comparison of the population genetic structure and diversity between a common (*Chrysemys p. picta*) and an endangered (*Clemmys guttata*) freshwater turtle. Diversity. 2019:11:99. doi: 10.3390/d11070099

[CIT0009] Bundy RE. New locality records of reptiles in New Mexico. Copeia. 1951:1951:314

[CIT0010] Collins G , Rosen JA. Frac water acquisition in the major U.S. Unconventional Oil and Gas Plays. In: Buono R, López Gunn E, McKay J, Staddon C, editors. Regulating water security in unconventional oil and gas. Cham, Switzerland: Springer; 2020:89–111. doi:10.1007/978-3-030-18342-4_5

[CIT0011] Cortés-Rodríguez X , Gabriela Borja-Martínez G, Vázquez-Domínguez E. Striking habitat reduction, decreased genetic diversity, and imperiled conservation of natural populations of *Terrapene coahuila*. Freshw Biol. 2021:66:842–858. doi: 10.1111/fwb.13681

[CIT0012] Dearen P. Bitter waters: the struggles of the Pecos River. Norman, Oklahoma, USA: University of Oklahoma Press; 2016

[CIT0013] Degenhardt WG , Painter CW, Price AH. Amphibians and reptiles of New Mexico. Albuquerque, New Mexico, USA: University of New Mexico Press; 1996

[CIT0014] Dixon JR. Amphibians and reptiles of Texas: with keys, taxonomic synopses, bibliography, and distribution maps. 3rd ed, Revised and Updated. College Station, Texas, USA: Texas A&M University Press; 2013

[CIT0015] Do C , Waples RS, Peel D, Macbeth GM, Tillett BJ, Ovenden JR. NeEstimator v2: re-implementation of software for the estimation of contemporary effective population size (Ne) from genetic data. Mol Ecol Resour. 2014:14:209–214 23992227 10.1111/1755-0998.12157

[CIT0016] Dunson WA , Seidel ME. Salinity tolerance of estuarine and insular Emydid turtles (*Pseudemys nelsoni* and *Trachemys decussata*). J Herpetol. 1986:20:237–245

[CIT0018] Ernst CH , Lovich JE. Turtles of the United States and Canada. Baltimore, Maryland, USA: John Hopkins University Press; 2009

[CIT0019] Evanno G , Regnaut S, Goudet J. Detecting the number of clusters of individuals using the software STRUCTURE: a simulation study. Mol Ecol. 2005:14:2611–2620 15969739 10.1111/j.1365-294X.2005.02553.x

[CIT0020] Fitak RR , Johnsen S. Green sea turtle (*Chelonia mydas*) population history indicates important demographic changes near the mid-Pleistocene transition. Mar Biol. 2018:165:110. doi: 10.1007/s00227-018-3366-3

[CIT0021] Forstner MR , Dixon JR, Guerra TM, Winters JM, Stuart JN, Davis SK. Status of US populations of the Big Bend slider (*Trachemys gaigeae*). In Hoyt CA, Karges J, editors. Proceedings of the Sixth Symposium on the Natural Resources of the Chihuahuan Desert Region; 2014 Oct 14–17; Fort Davis: Chihuahuan Desert Research Institute

[CIT0022] Fox EA , Wright AE, Fumagalli M, Vieira FG. *ngsLD*: Evaluating linkage disequilibrium using genotype likelihoods. Bioinformatics. 2019:35:3855–3856. doi: 10.1093/bioinformatics/btz200 30903149

[CIT0023] Galindo R , Wilson D, Caldwell CA. Geographic distribution of genetic diversity in populations of Rio Grande chub *Gila pandora*. Conserv Genet. 2016:17:1081–1091. doi: 10.1007/s10592-016-0845-2

[CIT0024] Gallego-García N , Caballero S, Shaffer HB. Are genomic updates of well-studied species worth the investment for conservation? A case study of the critically endangered Magdalena river turtle. J Hered. 2021:112:575–589 34628509 10.1093/jhered/esab063

[CIT0025] Georges A , Gruber B, Pauly GB, White D, Adams M, Young MJ, Kilian A, Zhang X, Shaffer HB, Unmack PJ. Genomewide SNP markers breathe new life into phylogeography and species delimitation for the problematic short-necked turtles (Chelidae: Emydura) of eastern Australia. Mol Ecol. 2018:27:5195–5213 30403418 10.1111/mec.14925

[CIT0026] Giermakowski JT , Pierce LJS. Geographic distribution: *Pseudemys gorzugi* (Rio Grande Cooter). Herpetol Rev. 2016:47:626

[CIT0027] Green RE , Braun EL, Armstrong J, Earl D, Nguyen N, Hickey G, Vandewege MW, St John JA, Capella-Gutiérrez S, Castoe TA, et al. Three crocodilian genomes reveal ancestral patterns of evolution among archosaurs. Science. 2014:346:1254449 25504731 10.1126/science.1254449 PMC4386873

[CIT0029] Hoagstrom CW. Causes and impacts of salinization in the lower Pecos River. Gt Plains Res. 2009:19:27–44

[CIT0030] Hoagstrom CW , Zymonas ND, Davenport SR, Propst DL, Brooks JE. Rapid species replacements between fishes of the North American plains: a case history from the Pecos River. Aquat Invasions. 2010:5:141–153

[CIT0031] Howell HJ , Legere RH, Holland DS, Seigel RA. Long-term turtle declines: protected is a verb, not an outcome. Copeia. 2019:107:493–501. doi: 10.1643/ch-19-177

[CIT0032] Inoue K , Lang BK, Berg DJ. Past climate change drives current genetic structure of an endangered freshwater mussel species. Mol Ecol. 2015:24:1910–1926 25782031 10.1111/mec.13156

[CIT0033] Jackson JT. Demography and population structure of a Rio Grande endemic emydid the Big Bend slider [Dissertation]. San Marcos, TX, USA: Texas State University; 2010

[CIT0034] Janes JK , Miller JM, Dupuis JR, Malenfant RM, Gorrell JC, Cullingham CI, Andrew RL. The K = 2 conundrum. Mol Ecol. 2017:26:3594–3602 28544181 10.1111/mec.14187

[CIT0035] Jensen R , Hatler W, Mecke M, Hart C. The influences of human activities on the waters of the Pecos Basin of Texas: a brief overview. Technical Report. SR-2006-03. College Station, Texas, USA: Texas Water Resources Institute; 2006.

[CIT0037] Johnston GR , Mitchell JC, Shemitz GA, Butt PL, Adler JM. Origin and structure of a large aggregation of Suwannee cooters (*Pseudemys concinna suwanniensis*) in a Florida spring. Chelonian Conserv Biol. 2018:17:54–62. doi: 10.2744/ccb-1290.1

[CIT0038] Karatayev AY , Burlakova LE, Miller TD, Perrelli MF. Reconstructing historical range and population size of an endangered mollusc: long-term decline of *Popenaias popeii* in the Rio Grande, Texas. Hydrobiologia. 2018:810:333–349

[CIT0039] Kimura M , Weiss GH. The stepping stone model of population structure and the decrease of genetic correlation with distance. Genetics. 1964:49:561–576 17248204 10.1093/genetics/49.4.561 PMC1210594

[CIT0040] Korneliussen TS , Albrechtsen A, Nielsen R. ANGSD: Analysis of next generation sequencing data. BMC Bioinf. 2014:15:1–13. http://www.biomedcentral.com/1471-2105/15/356 10.1186/s12859-014-0356-4 PMC4248462 25420514

[CIT0041] Korneliussen TS , Moltke I, Albrechtsen A, Nielsen R. Calculation of Tajima’s D and other neutrality test statistics from low depth next-generation sequencing data. BMC Bioinf. 2013:14:289. http://www.biomedcentral.com/1471-2105/14/289 10.1186/1471-2105-14-289 PMC4015034 24088262

[CIT0042] Legler J , Vogt RC. The turtles of Mexico: land and freshwater forms. Los Angeles, California, USA: University of California Press; 2013

[CIT0043] Letter AW , Waldon KJ, Pollock DA, Mali I. Dietary habits of Rio Grande cooters (*Pseudemys gorzugi*) from two sites within the Black River, Eddy County, New Mexico, USA. J Herpetol. 2019:53:204–208. doi: 10.1670/18-057

[CIT0044] Li H , Durbin R. Fast and accurate long-read alignment with Burrows-Wheeler transform. Bioinformatics. 2010:26:589–595 20080505 10.1093/bioinformatics/btp698 PMC2828108

[CIT0045] Liu X , Fu Y-X. Exploring population size changes using SNP frequency spectra. Nat Genet. 2015:47:555–559 25848749 10.1038/ng.3254 PMC4414822

[CIT0046] Liu X , Fu Y-X. Author correction: Stairway Plot 2: demographic history inference with folded SNP frequency spectra. Genome Biol. 2020:21:305 33357235 10.1186/s13059-020-02243-5 PMC7767121

[CIT0047] Lovich JE , Ennen JR. A quantitative analysis of the state of knowledge of turtles of the United States and Canada. Amphib-Reptilia. 2013:34:11–23. doi: 10.1163/15685381-00002860

[CIT0048] Lovich JE , Ennen JR, Agha M, Gibbons JW. Where have all the turtles gone, and why does it matter? BioScience. 2018:68:771–781. doi: 10.1093/biosci/biy095

[CIT0049] MacLaren AR , Sirsi S, Foley DH, Forstner MRJ. *Pseudemys gorzugi* (Rio Grande cooter). Long distance dispersal. Herpetol Rev. 2017:48:180–181

[CIT0050] Mahan LB , Bassett LG, Duarte A, Forstner MRJ, Mali I. Effects of salinization on the occurrence of a long-lived vertebrate in a desert river. Sci Rep. 2022:12:15907. doi: 10.1038/s41598-022-20199-3 36151273 PMC9508222

[CIT0051] Mali I , Duarte A, Forstner MRJ. Comparison of hoop-net trapping and visual surveys to monitor abundance of the Rio Grande cooter (*Pseudemys gorzugi*). PeerJ. 2018:6:e4677. doi: 10.7717/peerj.4677 29770271 PMC5951123

[CIT0052] McGaugh SE. Comparative population genetics of aquatic turtles in the desert. Conserv Genet. 2012:13:1561–1576. doi: 10.1007/s10592-012-0403-5

[CIT0053] Meisner J , Albrechtsen A. Inferring population structure and admixture proportions in low-depth NGS data. Genetics. 2018:210:719–731. doi: 10.1534/genetics.118.301336 30131346 PMC6216594

[CIT0054] New Mexico Department of Game and Fish. Threatened and endangered species of New Mexico, 2020 biennial review. Santa Fe, New Mexico, USA: Management and Fisheries Management Divisions; 2020

[CIT0056] Osborne MJ , Portnoy DS, Fields AT, Bean MG, Hoagstrom CW, Conway KW. Under the radar: genetic assessment of Rio Grande shiner (*Notropis jemezanus*) and Speckled chub (*Macrhybopsis aestivalis*), two Rio Grande basin endemic cyprinids that have experienced recent range contractions. Conserv Genet. 2021:22:187–204. doi: 10.1007/s10592-020-01328-9

[CIT0082] Parham JF , Papenfuss TJ, Sellas AB, Stuart BL, Simison WB. Genetic variation and admixture of red-eared sliders (Trachemys scripta elegans) in the USA. *Mol**Phylogenetics**Evol*. 2020:145:106722. doi: 10.1016/j.ympev.2019.106722 31874235

[CIT0057] Pease AA , Delaune KD. Dried and salted: cumulative impacts of diminished flows and salinization on Lower Pecos River food webs. *Proceedings of the Desert Fishes Counil Special Publication*. 2021: 2-19

[CIT0058] Peterson BK , Weber JN, Kay EH, Fisher HS, Hoekstra HE. Double digest RADseq: an inexpensive method for de novo SNP discovery and genotyping in model and non-model species. PLoS One. 2012:7:e37135. doi: 10.1371/journal.pone.0037135 22675423 PMC3365034

[CIT0059] Petkova D , Novembre J, Stephens M. Visualizing spatial population structure with estimated effective migration surfaces. Nat Genet. 2016:48:94–100 26642242 10.1038/ng.3464 PMC4696895

[CIT0060] Pierce LJS , Stuart NJ, Ward JP, Painter CW. *Pseudemys gorzugi* Ward 1984–Rio Grande Cooter, Western River Cooter, Tortuga de Oreja Amarilla, Jicotéa del Rio Bravo in conservation biology of freshwater turtles and tortoises: A Compilation Project of the IUCN/SSC Tortoise and Freshwater Turtle Specialist Group (eds. Rhodin, A. G. J. *et al*.). Chelonian Res Monog. 2016:5:100.1–100.12. doi: 10.3854/crm.5.100.gorzugi.v1.2016

[CIT0061] Reid BN , Pinsky ML. Simulation-based evaluation of methods, data types and temporal sampling schemes for detecting recent population declines. Integr Comp Biol. 2022:62:1849–1863 36104155 10.1093/icb/icac144 PMC9801984

[CIT0062] Rhodin AGJ , Iverson JB, Bour R, Fritz U, Georges A, Shaffer HB, van Dijk PP]. Turtles of the world: annotated checklist and atlas of taxonomy, synonymy, distribution, and conservation status. In: Rhodin AGJ, Iverson JB, van Dijk PP, Stanford CB, Goode EV, Buhlmann KA, Mittermeier RA, editors. Conservation biology of freshwater turtles and tortoises: a compilation project of the IUCN/SSC Tortoise and Freshwater Turtles Specialist Group. 9th ed. Chelonian Research Monographs 8; 2021. p. 1–472. doi:10.3854/crm.8.checklist.atlas.v9.2021

[CIT0063] Rochette NC , Rivera-Colón AG, Catchen JM. Stacks 2: analytical methods for paired-end sequencing improve RADseq-based population genomics. Mol Ecol. 2019:28:4737–4754 31550391 10.1111/mec.15253

[CIT0064] Scanlon BR , Reedy RC, Wolaver BD. Assessing cumulative water impacts from shale oil and gas production: Permian Basin case study. Sci Total Environ. 2022:811:152306 34906580 10.1016/j.scitotenv.2021.152306

[CIT0065] Seidel ME. Osmoregulation in the turtle Trionyx spiniferus from brackish and fresh water. Copeia 1975:1975:124–128

[CIT0066] SEMARNAT [Secretaríade Medio Ambiente y Recursos Naturales]. NORMA Oficial Mexicana NOM-059-SEMARNAT-2010, Protección ambiental–Especies nativas de México de flora y fauna silvestres–Categorías de riesgo y especificaciones para su inclusión, exclusión o cambio–Lista de especies en riesgo. Diario Oficial de la Federación 2010:2

[CIT0067] Shaffer HB , Minx P, Warren DE, Shedlock AM, Thomson RC, Valenzuela N, Abramyan J, Amemiya CT, Badenhorst D, Biggar KK, et al. The western painted turtle genome, a model for the evolution of extreme physiological adaptations in a slowly evolving lineage. Genome Biol. 2013:14:R28. doi: 10.1186/gb-2013-14-3-r28 23537068 PMC4054807

[CIT0068] Sirsi S. The impact of dispersal assessment methods on the resulting management interpretations of endangered species stewardship [Dissertation]. San Marcos Texas, USA:Texas State University; 2021

[CIT0069] Skotte L , Korneliussen TS, Albrechtsen A. Estimating individual admixture proportions from next generation sequencing data. Genetics. 2013:195:693–702. doi: 10.1534/genetics.113.154138 24026093 PMC3813857

[CIT0070] Suriyamongkol T , Mali I. Aspects of the reproductive biology of the Rio Grande cooter (*Pseudemys gorzugi*) on the Black River, New Mexico. Chelonian Conserv Biol. 2019:18:187–194. doi: 10.2744/ccb-1385.1

[CIT0071] Suriyamongkol T , Ortega-Berno V, Mahan LB, Mali I. Using stable isotopes to study resource partitioning between red-eared slider and Rio Grande cooter in the Pecos River watershed. Ichthyol Herpetol. 2022:110:96–105. doi: 10.1643/h2021023

[CIT0072] Suriyamongkol T , Waldon KJ, Ortega-Berno V, Mahan LB, Creswell MA, Mali I. Geographic distribution: *Pseudemys gorzugi* (Rio Grande cooter). Herpetol Rev. 2020:51:536–537

[CIT0073] Texas Parks & Wildlife Department. Species account: the Rio Grande River Cooter (*Pseudemys gorzugi*). In: Bender S, Shelton S, Bender K, Kalmbach A, editors. Texas Comprehensive Wildlife Conservation Strategy 2005–2010. Austin, Texas, USA: Nongame Division; 2012. p. 1075–7076

[CIT0074] Tyers M. riverdist: River Network Distance Computation and Applications. R package version 0.16.2. 2023 [accessed 2023 Dec 12]. <https://CRAN.R-project.org/package=riverdist>.

[CIT0075] U.S. Fish and Wildlife Service. Species Status Assessment Report for Rio Grande cooter (*Pseudemys gorzugi*). Version 1.0. Austin, Texas: U.S. Fish and Wildlife Service; 2021

[CIT0076] U.S. Fish and Wildlife Service. Endangered and threatened wildlife and plants; three species not warranted for listing as endangered or threatened species. Fed Regist. 2022:87:14227–14232

[CIT0077] van Dijk PP. *Pseudemys gorzugi* (errata version published in 2016). The IUCN Red List of Threatened Species 2011:2011:e.T18459A97

[CIT0078] Viera FG , Lasalle F, Korneliussen TS, Fumagalli M. Improving the estimating of genetics distances from next-generation sequencing data. Biol J Linn Soc. 2015:117:139–149. doi: 10.1111/bij.12511

[CIT0079] Walker J , Dornak DN, Schlandt A. A path forward for the Pecos River watershed protection plan. Report No. 2021-04. San Marcos, TX: Texas State University; 2021

[CIT0080] Williams AP , Cook BI, Smerdon JE. Rapid intensification of the emerging southwestern North American megadrought in 2020–2021. Nat Clim Change. 2022:12:232–234. doi: 10.1038/s41558-022-01290-z

[CIT0081] Xie Q , Rong C, Li X, Lai N, Chen T, Li Q, Su S. SNP discovery in *Mauremys reevesii* and *M. sinensis* using restriction site-associated DNA sequence (RAD-seq). Conserv Genet Resour. 2019:12:389–393

