# Supplementary material for: Patterns of genetic divergence in the Rio Grande cooter (Pseudemys gorzugi), a riverine turtle inhabiting an arid and anthropogenically modified system
Source: J Hered. 2024 Feb 19;115(3):253–61. doi: 10.1093/jhered/esae011 (PMC11081133; doi:10.1093/jhered/esae011)
Supplement: esae011_suppl_Supplementary_Figures_1-7 [file esae011_suppl_supplementary_figures_1-7.docx]

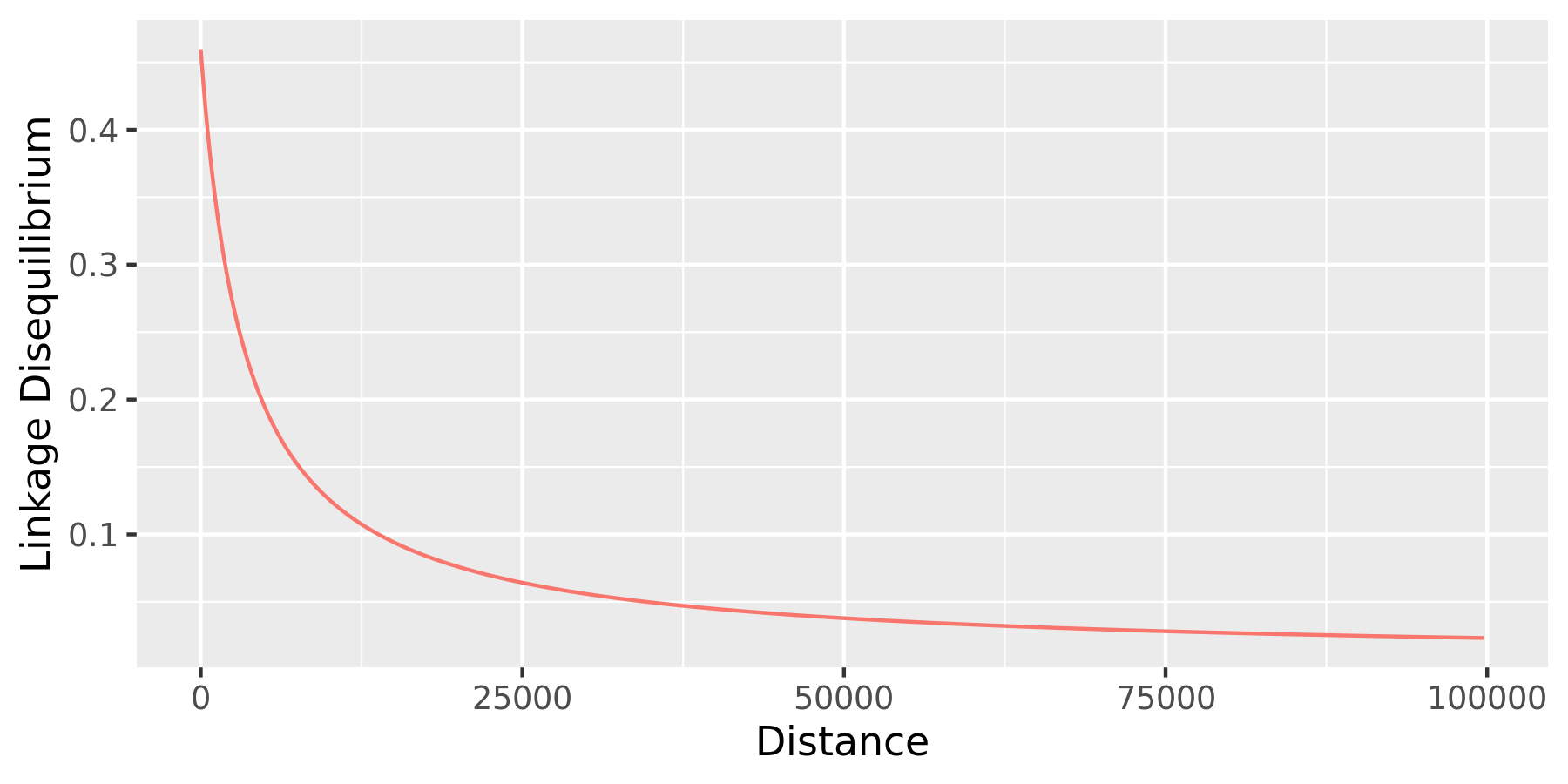


Supplemental Figure 1. Linkage disequilibrium decay for sites between 0 and 100 kb apart computed by fit_LDdecay.R provided with ngsLD.


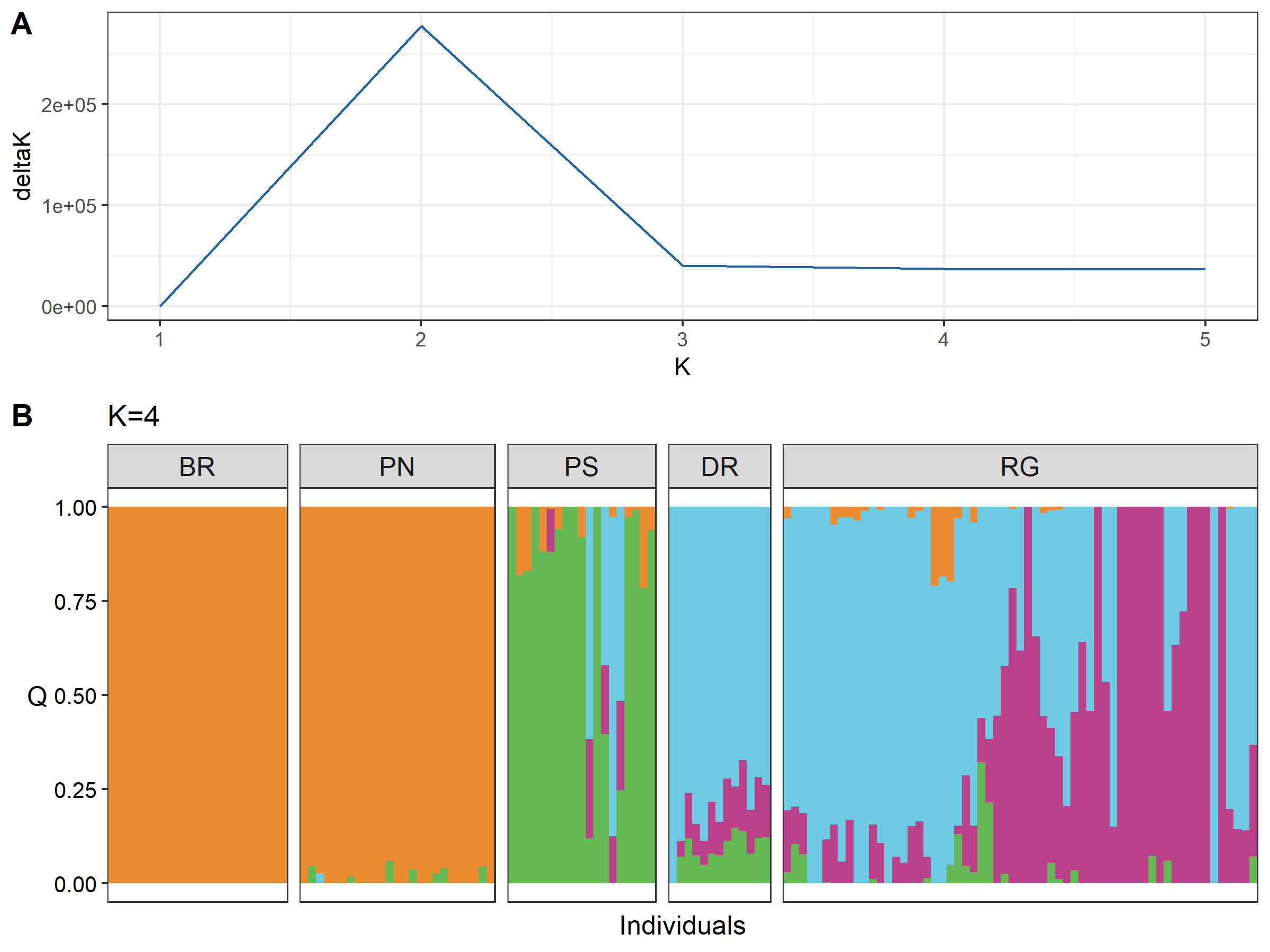


Supplemental Figure 2. A) Change in log likelihood of K from K =1 – 5. B) Structure plots for K = 3.


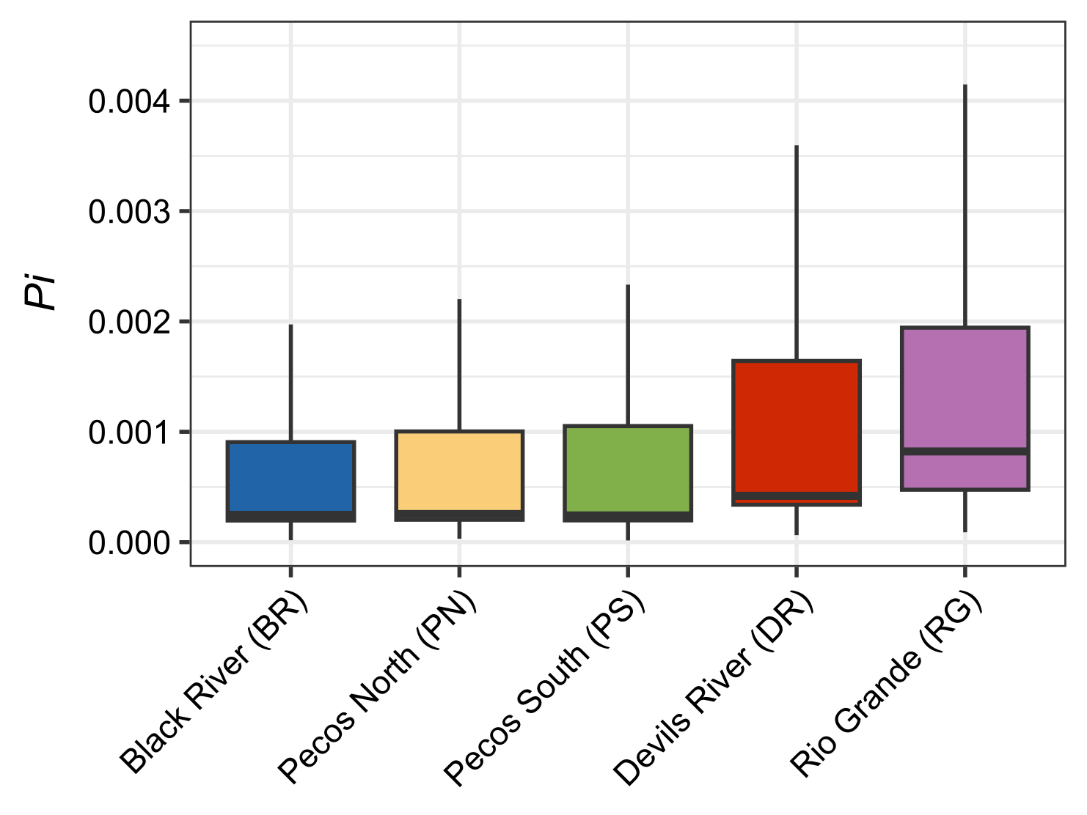


Supplementary Figure 3. Nucleotide diversity (*Pi*) distribution generated from 10kb windows for each *a priori* population.


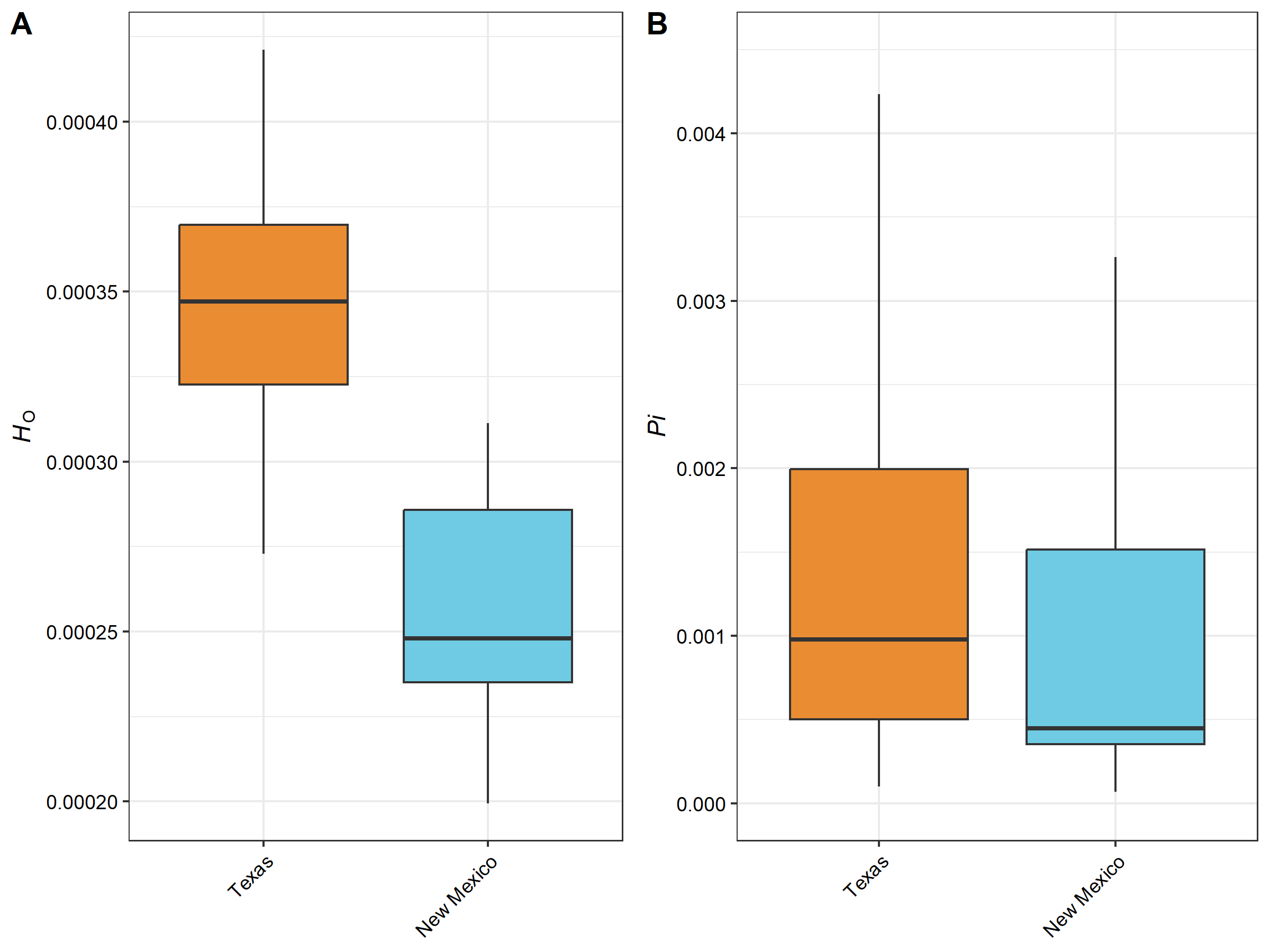


Supplemental Figure 4. A) Observed heterozygosity and B) *Pi* comparisons between the Texas (RG + DR) and New Mexico (PN + BR) populations.


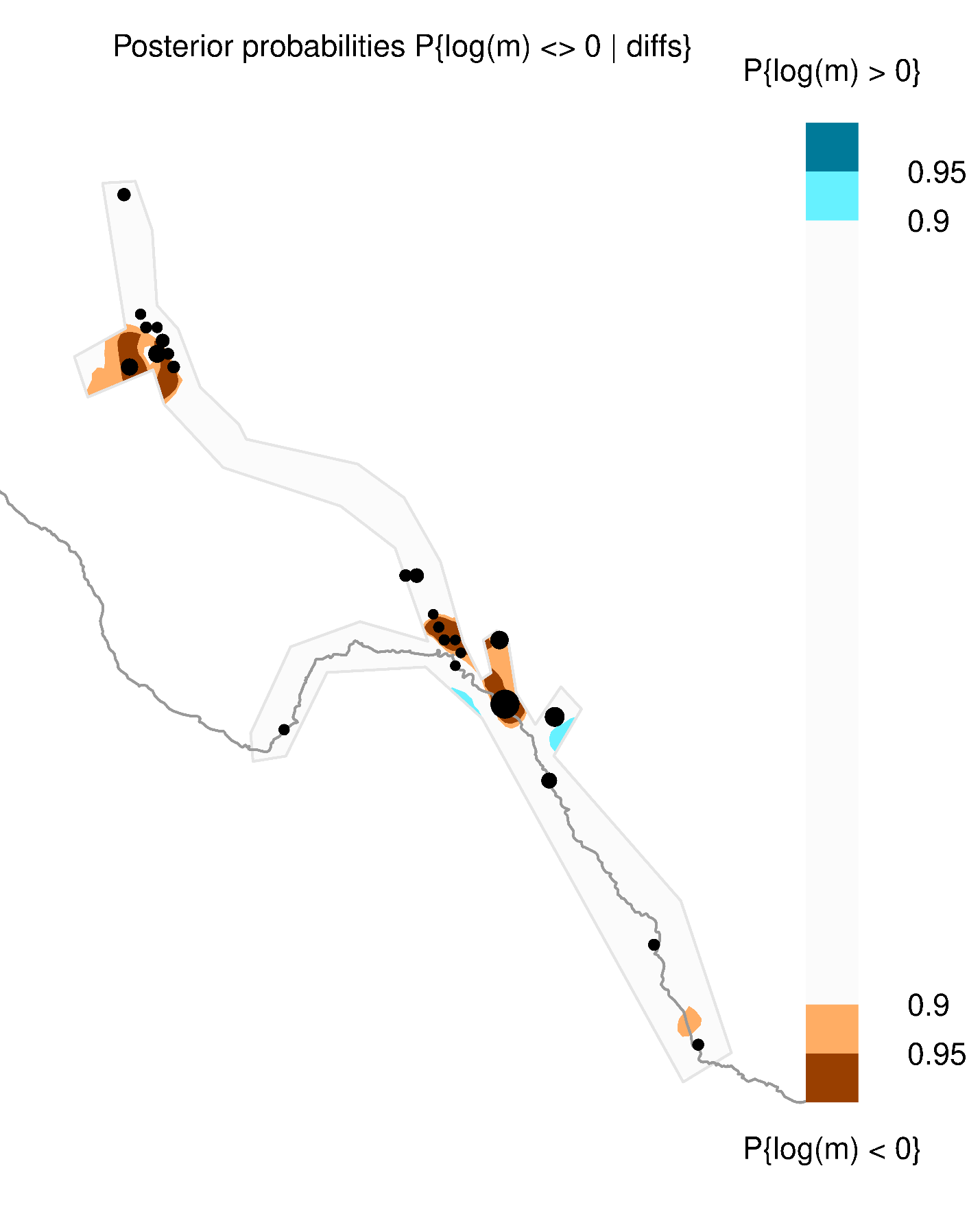


Supplemental Figure 5. Where effective migration posterior probabilities were greater than 0.9.


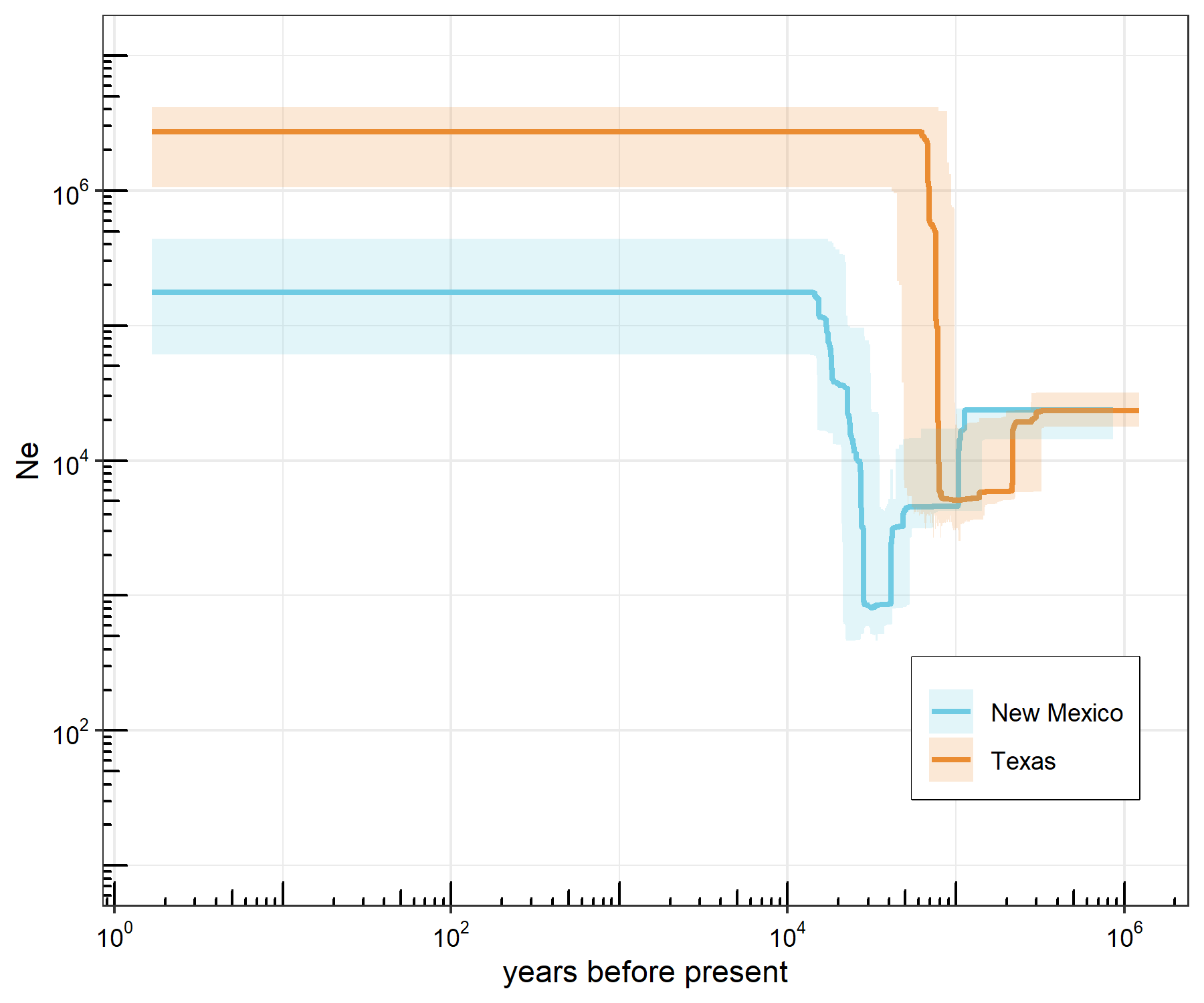


Supplemental Figure 6. Change in *N*_e_ over the past million years estimated by Stairway Plot 2. Dark lines represent the median *N*_e_ and lighter shades reflect the 95% confidence interval


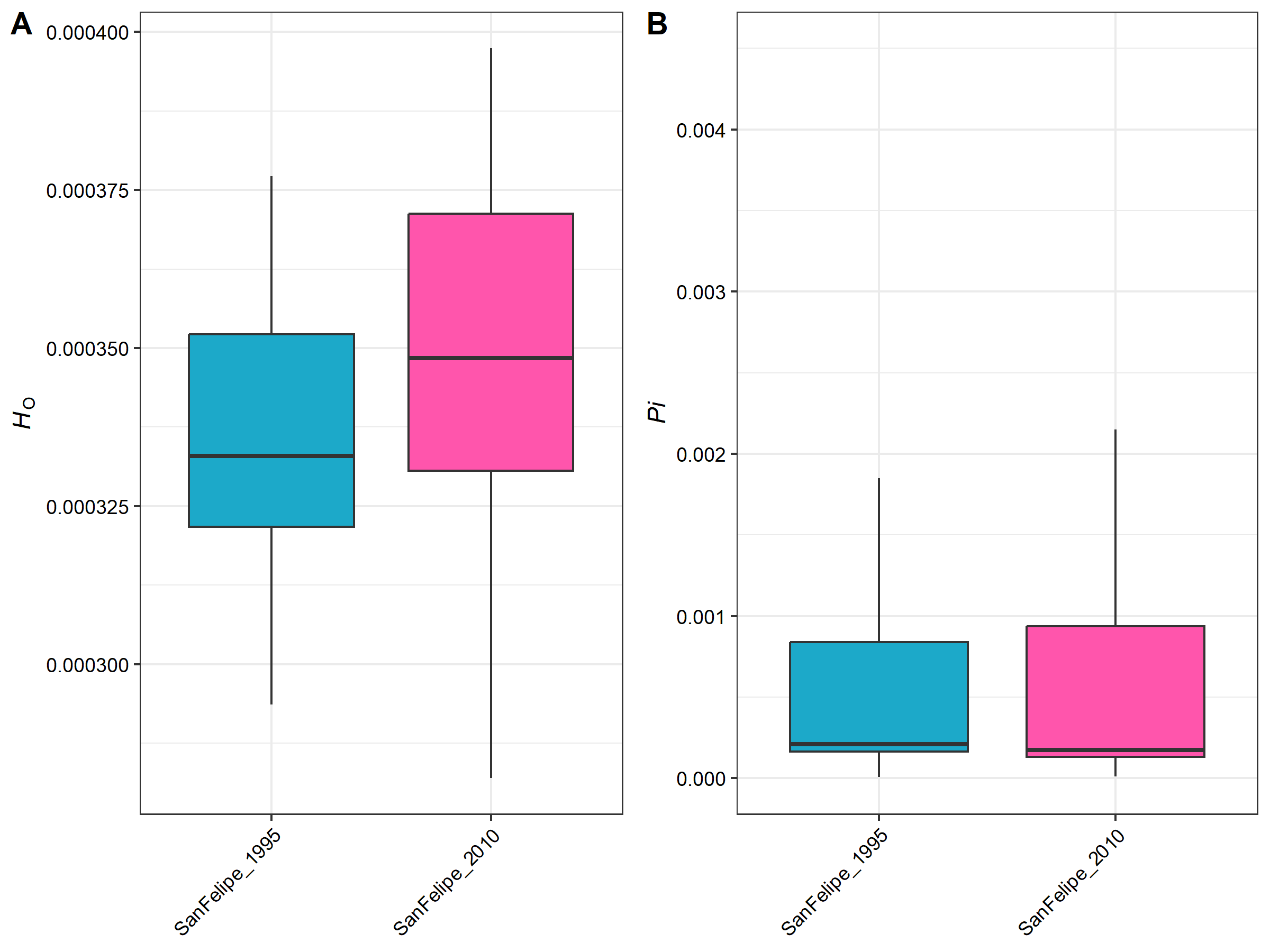


Supplemental Figure 7. A) The observed heterozygosity and B) nucleotide diversity comparison between historic and modern samples collected from San Felipe springs.
