# Supplementary material for: Patterns of genetic divergence in the Rio Grande cooter (Pseudemys gorzugi), a riverine turtle inhabiting an arid and anthropogenically modified system
Source: J Hered. 2024 Feb 19;115(3):253–61. doi: 10.1093/jhered/esae011 (PMC11081133; doi:10.1093/jhered/esae011)
Supplement: esae011_suppl_Supplementary_Table_1 [file esae011_suppl_supplementary_table_1.zip › Suppl_table 1/Supplementary Table 1.docx]

Supplementary Table 1. Sample meta data including the sample ID, state and county of capture, sex if applicable, museum collection information and *a priori* population designation. TNHC = Biodiversity Collections, The University of Texas at Austin; TSU = Texas State University; NCSU = North Carolina State University.
